# Progressive Trends on the Biomedical Applications of Metal Organic Frameworks

**DOI:** 10.3390/polym14214710

**Published:** 2022-11-03

**Authors:** Gaurav Awasthi, Sahil Shivgotra, Shibyendu Nikhar, Subramanian Sundarrajan, Seeram Ramakrishna, Pawan Kumar

**Affiliations:** 1 Materials Application Research Laboratory, Department of Nano Sciences and Materials, Central University of Jammu, Jammu 181143, India; 2 NUS Centre for Nanotechnology and Sustainability, Department of Mechanical Engineering, National University of Singapore, Singapore 11758, Singapore; 3 Department of Prosthodontics, Saveetha Dental College and Hospitals, Saveetha Institute of Medical & Technical Sciences, Saveetha University, Chennai 600077, India

**Keywords:** MOF technology, biomedical applications, biomolecules, drug, toxicity

## Abstract

Novel materials have been developed because of technological advancements combined with material research. Metal-organic frameworks (MOF) technology has been investigated for biomedical applications in this line. Nonetheless, as our team has learned from current literature, selecting metal ions/organic linkers, synthesis techniques, water stability/solubility, toxicity, and the possibility of biomolecules/drugs (enzyme, protein, DNA/RNA, and antibodies, among others) tagging/conjugation are the major challenges/factors. These issues/factors have an impact on MOFs’ performance in biomedical applications, and they also raise a lot of doubts about its real-time biological utility in the near future. We targeted a comprehensive review on the MOFs for biomedical applications to keep these considerations in mind. The evolution of MOF technology is based on their interesting features such as biological or pharmacological activity, biocompatibility, limited toxicity, and particular host–guest interactions, as well as environmental friendliness. In this paper, we have summarized the state-of-the-art progress pertaining to MOFs’ biomedical applications such as biosensing, biomedical, and drug delivery applications in this field that is still very new.

## 1. Introduction

MOFs, or metal-organic frameworks, are a new form of a porous coordination polymer. Due to their considerable amount of diversity in their structure and increased surface area, these exciting materials have a significant impact on a various field such as academics, industries, and so on. Today several companies like BASF (Sigma-Aldrich, St. Louis, MO, USA, 2020), NuMat Technologies (NuMat Technologies, Skokie, IL, USA, 2020), MOFapps (MOFapps, Oslo, Norway 2020), framergy (Framergy, Wilmington, NC, USA, 2020), metalorganic-frameworks.EU (Materials Center, Leoben, Austria, 2020), ImmondoTech, and MOF Technologies (MOF Technologies, Belfast, UK, 2020) produce MOFs on an industrial scale. MOFs have a diverse range of applications, namely, separation, gas storage, sensing, proton conduction, catalysis, etc. [1]. Due to their unique properties as hybrid composite systems, MOFs have been thoroughly investigated in a diverse application, particularly in biological fields.

MOFs have also been referred to as materials made of organic–inorganic hybrids, coordination polymers, porous coordination networks, and metal organic polymers in the literature. In the area of nanoporous material research, MOFs were among the most alluring substances. MOFs are smart alternatives to conventional nanoporous materials in a wide range of scientific and industrial fields due to their excellent combination of high porosity, absence of inaccessible bulk volume, incredibly wide range of topologies and pore sizes, vast surface areas, and a wide range of potential structure viability. The synthesis of MOFs based on the reticular design concept in late 1999 sparked interest in this field even though they have been known about since 1965 [2].

Although around 25,000 MOFs have been synthesized and characterized, BioMOFs is a new subclass of MOFs composed of biomolecules as linkers [3]. They are defined as the MOF, which constitutes at least one biomolecule as a linker [4]. The common biological organic linkers include amino acids, proteins, nucleobases, polypeptides, saccharides, and cyclodextrins [4]. Often, it has been observed that some auxiliary linkers like dicarboxylates can also be used as organic linkers. BioMOFs are either two-dimensional or three-dimensional structures with excellent porosity generated using coordination bonding between transition metal ions and biomolecules rather than a conventional organic linker [5]. The potential of BioMOFs have been demonstrated for several biological and medical applications such as sensing, imaging, and drug delivery [5]. To maintain a good host–guest response towards the biological systems, BioMOFs should necessarily be biocompatible [3,5]. To address the fundamental performances and characteristics of MOFs for biological applications, there are a variety of methods that may be applied, including (i) covalent bonding, (ii) non-covalent attachment, (iii) polymer coordination, and (iv) encapsulation. For example, Cu and Eu-TCA (H_3_TCA = tricarboxytriphenyl amine) MOFs have a luminescent property to detect (highly toxic and carcinogenic) in an aqueous solution and living cells [6]. The first context of MOFs in the biological application was being used as vectors for drug delivery; their large pore volumes, and their diverse structure makes MOFs a suitable candidate for a drug delivery system (DDS) [5,7,8]. Pore chemistry, crystal size, and stable framework inflect characteristics; controlled release rates, biocompatibility, and loading capacities ensure MOFs as carriers for a drug in recent reports. For instance, MOFs have been used as encapsulated macromolecules, which can post synthetically infiltrated micro peroxidase MP-11 and cytochrome-C into Tb-based MOFs [9,10]. It has been reported that the potential application of MOFs can be achieved by precipitation of a porous framework by enzymes and proteins in areas such as bio catalysis and bio-banking [11,12]. Water is a non-toxic medium, and it is an essential and important solvent system for biological application. Non-toxicity, biocompatibility, green recyclable, and low cost are important features for MOF technology. On the other hand, water stability and solubility have been majorly considered for membrane separation, adsorption, sensing, catalysis, drug delivery, and imaging.

The current review highlights recent developments in metal-organic frameworks, which are promising nanocarriers for the delivery of drugs and the identification of diseases in the biomedical field. First, a basic overview of MOFs and their classification is addressed. Then, for diverse diseases, recent diagnostics and applications of MOFs (such as in biosensing, biomedical imaging, and in DDS) are illustrated. In order to prepare the way for further investigation of MOFs as innovative theranostic systems for biomedical applications, conclusions are formed and challenges are compiled at the end of this review.

## 2. Classification of MOFs

**Figure 1:**
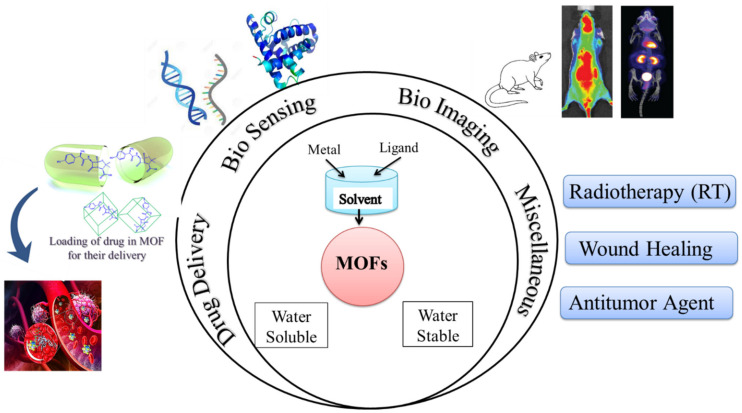
A-line diagram showing the MOF types on the basis of water solubility and their bio application with their examples and methods (adapted from [12,13,14,15,16]).

Based on solubility, MOFs have been classified into two primary classes of MOFs (Figure 1), i.e., (a) Water-stable MOFs and (b) Water-soluble MOFs.

### 2.1. Water-Soluble MOFs

These types of MOFs have low thermodynamic and kinetic stabilities. They showed faster hydrolysis processes due to the high water ligand exchange rate [13]. Water-soluble MOFs also have a wide role in various aspects. The addition of strong polar groups such as -OH, -COOH, and -NH_2_ on MOF surface transforms it from non-polar to polar, thereby enhances membrane hydrophilicity [14]. MOFs are generally stable in water. However, modern medical and drug release approaches are required to gain the water-solubility phenomenon. The solubility of MOFs promptly depends on their crystallinity. It has been found that MOFs having lower crystallinity are generally water-soluble. Since hydrophilicity and hydrophobicity is related with the contact angle, a contact angle less than 90 °C indicates the hydrophilicity of compounds [15]. The addition of a strong polar group increased the polarity of MOF and decreased the contact angle [16], and it enhanced the hydrophilicity.

### 2.2. Water-Stable MOFs

The scientific community is interested in water-stable MOFs because the majority of industrial processes contain some level of water or moisture in various forms of preparation, storage, transportation, and applications. Water-stable MOFs are stable in the presence of water to make them more viable commercially and industrially in water. Water stability is the most important factor for real-world applications because our environment has a lot of water as moisture or many other forms to degrade them and decrease their functional activities like adsorption, degradation, etc. Large-scale production of MOFs is still hard because of their different properties, different ways of making them, and quality control, high production cost, lack of standardization, etc. [17]. Some commercially available water-stable MOFs are Basolite^R^F300 (Fe-BTC), Basolite^R^A100 (MIL-53(Al)), and Basolite^R^Z1200 (ZIF-8), etc., which are manufactured by Sigma-Aldrich [17].

**Figure 2:**
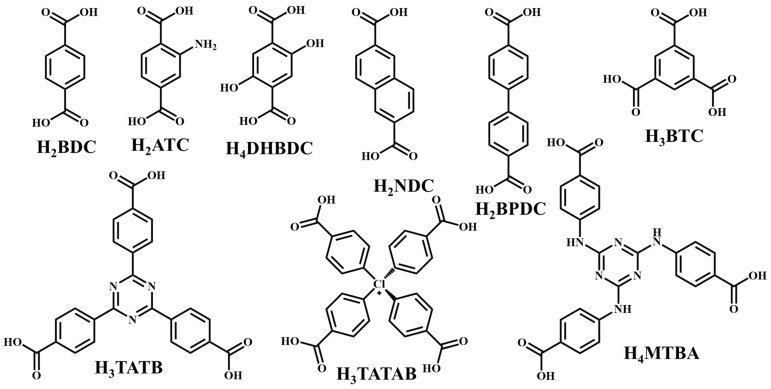
Chemical structures of some carboxylate linkers; H_2_BDC = 1,4-benzenedicarboxylic acid, H_2_ATC = 2-aminoterephthalic acid, H_2_NDC = 2,6-naphthalenedicarboxylic acid, H_2_BPDC = 4,4′-biphenyldicarboxylic acid, H_4_DHBDC= 2,5-Dihydroxy-1,4-benzenedicarboxylic acid, H_3_TATAB = 4,4′, 4″-s-triazine-1,3,5-triyltri-p-aminobenzoic acid, H_2_TATB = 4,4′,4″-s-triazine-2,4,6-triyl-tribenzoic acid, H_4_MTBA = methanetetra (4-benzoic acid) [55,56].

**Figure 3:**
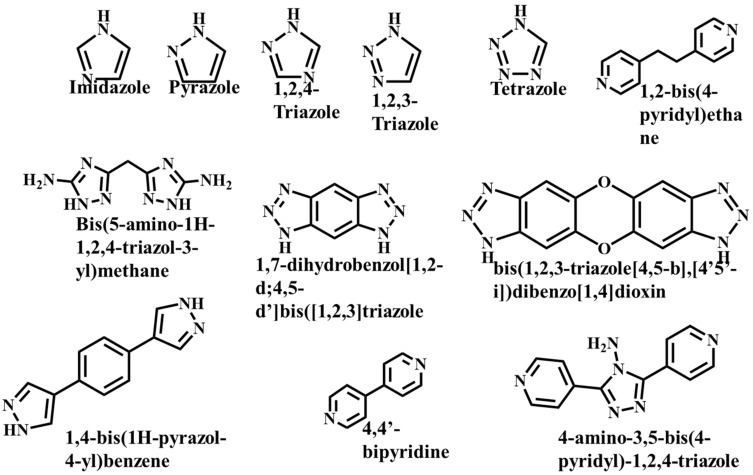
Chemical Structure of some azolate-based and N-doner-based linkers [57].

It is often possible to determine whether the MOF structure is either stable or unstable in the water. Generally, it is discovered by comparing the chemical properties of pre- and post-exposure samples. After the comparison of powder X-ray diffraction (PXRD) data and gas adsorption isotherm base BET surface area, they could present a convincing argument for whether the crystallinity or structural porosity of the MOF were lost or not after coming into contact with water. Typically, water exposure causes ligand displacement, structural disintegration, and phase shifts in MOFs. Suitable strength is required for a water-stable MOF structure to withstand over water molecule invasions; along with the ensuing loss of crystallinity and overall porosity, water-stable MOF building blocks should be suitably strong. These structures with high stability typically include strong coordination bonds (thermodynamic stability) to prevent the metal-ligand connections from being broken during the hydrolysis reaction. Therefore, it is crucial to accurately determine how water molecules affect the fundamental MOF characteristics (such as structural stability and metal-ligand coordination), especially for the synthesis of water-stable MOFs (WMOFs) [18] [Table 1]. In accordance with Pearson’s HSBA (hard-soft acid-base) principle, the ligands based on carboxylates are known as hard bases, those generate stable MOFs in addition to high-valent metal ions, e.g., Al^3+,^ Ti^4+^, Fe^3+^, Cr^3+^, and Zr^4+^. Moreover, soft azolate ligands (triazolates, imidazolates, tetrazolates, and pyrazolates) and soft divalent metal ions (Mn^2+^, Cu^2+^, Zn^2+^, Ni^2+^, and Ag^2+^) can also form stable MOFs. Zeolitic imidazolate frameworks (ZIFs) are the most well-known examples, composed of Zn^2+^ and imidazolate linkers [19]. Chemical structures of some carboxylate- and azolate-based linkers are given in Figure 2 and Figure 3 [12,13,14,15,16], respectively.

**Table 1:** Recently reported water-stable MOFs for biomedical applications.

S. No.	MOF	Metal	Ligand	Use	Targeting Agent	References
1	FMOF-1	Ag	3,5-bis(trifluoromethyl)-1,2,4-triazolate	Adsorption	CO_2_, Water	[20]
2	Al_2_(OH)_2_TCPP-Co	Al	4,4′,4″,4‴-(porphyrin-5,10,15,20-tetrayl) tetrabenzoate	Catalysis	Reduction of carbon dioxide	[20]
3	CAU-1	Al	1,4-benzenedicarboxylate (terephthalate)	Organic Adsorption	Nitrobenzene	[21]
4	CAU-10	Al	1,3-benzene dicarboxylic acid	Sensing applications	Water	[22]
5	MIL-121	Al	1,2,4,5-benzene tetracarboxylic acid, pyromellitic acid	Sensing applications Adsorption conductivity	Hippuric acid, Cu(II) Li and Na	[22]
6	MIL-96	Al	1,3,5,-bezenetricarboxylate	Adsorption	Fluoride, CO_2_, NO_2_, p-HBA	[23,24]
7	Co-ZiF-9	Co	Benzimidazole	Catalysis	Oxygen evolution reaction	[22]
8	ZIF-67	Co	2-methylimidazole	Adsorption	Benzotriazole, CO_2_, Methylene blue, Neutral rhodamine, Methyl orange	[25]
9	MIL-101	Cr/Al	(O_2_C)-C_6_H_4_-(CO_2_)	Organic Adsorption, Catalyst, Separation	Ukraine, cyanosilylation reaction, Knoevenagel condensation, CO_2_/CH_4_ Separation	[26,27]
10	Cu(i)-MOF	Cu	1-benzimidazolyl-3,5-bis(4-pyridyl) benzene	Sensing applications	Water and formaldehyde	[22]
11	HKUST-1	Cu	1,3,5-benzenetricarboxylate	Sensing applications, Adsorption	Water NH_3_, CO_2_, NO_2_	[28,29]
12	PCP-33	Cu	3,5-bis(2H-tetrazol-5-yl)-benzoic acid	Gas Adsorption	C_2_H_2_	[30]
13	MIL-68	Fe	1,4-benzenedicarboxylate(terephthalate)	Organic Adsorption, Catalyst	Phenol, reduction of Cr(VI), condensation between alcohols and o-aminothio-phenols	[31,32]
14	PCF-1	In	4′-phosphonobiphenyl-3,5-dicarboxylate	Sensing applications	Methylviologen and Cu^2+^ ions	[33]
15	AEMOF-1	Mg	2,5-dihydroxy-terephthalic acid; N,N-dimethylacetamide	Sensing applications	Water	[34]
16	MOF-74/PES	Mg	2,5-dioxide-1,4-benzenedicarboxylate	Membrane Separation	Ultrafiltration (BSA rejection)	[35]
17	PCMOF-10	Mg	2,5-dicarboxy-1,4-benzene-diphosphonic aci	Proton conduction		[22]
18	MAF-X25 ox	Mn	1H,5H-benzo(1,2-d:4,5-d0)bistriazole	Gas Adsorption	CO_2_	[36]
19	Na-HIPAA	Na	Hydroxyphosphonoacetate	Proton conduction		[22]
20	BFMOF-1	Pb	N,N-dimethylacetamide	Sensing applications	H_2_S	[37]
21	Tb-DSOA	Tb	2,2′-disulfonate-4,4′-oxydibenzoic acid	Proton conduction		[22]
22	NH2-MIL-125(Ti)	Ti	(O_2_C)-C_6_H_4_-(CO_2_)	Sensing applications, Adsorption	Water CO_2_, isoprene	[38,39]
23	MAF-6	Zn	2-ethylimidazolate	Organic Adsorption	Methanol, ethanol, benzene	[40]
24	ZIF-7	Zn	Benzimidazole	Membrane Separation, Adsorption	H_2_/CO_2_ Separation, Ethane, CH_4_, CO_2_	[41]
25	ZIF-8	Zn	2-methylimidazole	Organic Adsorption, Sensing, Proton conduction Membrane Separation	Phthalic acid, Water sensing, Furfural	[22,42]
26	ZIF-90	Zn	2-carboxaldehyde imidazolate	Membrane Separation Adsorption and Heat storage	H_2_/CH_4_ and H_2_/CO_2_ Separation Hg(II)	[43]
27	MENU-500	Zn, Mo	Benzene tribenzoate; Tetrabutylammonium Ion	Catalysis	Hydrogen evolution reaction	[44]
28	NU-1000	Zr	1,3,6,8-tetrakis(p-benzoic acid)pyrene	Bioimaging		[45]
29	NU-1100	Zr	4-[2-[3,6,8-tris [2-(4-carboxyphenyl)-ethynyl]-pyren-1-yl]ethynyl]-benzoic acid	Gas Adsorption	H_2_, CH_4_	[46]
30	PCN-222	Zr	(Tetrakis(4-carboxyphenyl)porphyrin	Catalysis	Cycloaddition Reactions	[46]
31	UiO-66	Zr	Benzene-1,4-dicarboxylic acid	Drug Delivery, Gas Absorption, Membrane separation	Pulmonary drugs	[47,48]
32	UiO-67	Zr	Biphenyldicarboxylate	Catalyst Organic Adsorption	Friedel–Crafts Alkylation Toluene	[49,50]
33	CdEDDA	Cd	EDDA	Sensing applications	Hg(II)	[22]
34	Co-MOF-74	Co	2,5-dioxide-1,4-benzenedicarboxylate	Adsorption and catalyst	CO_2_, Cycloaddition reaction	[51]
35	FIR-54	Zn	tris(4-(1H-imidazole-1-yl)phenyl)amine and Dimethylformamide	Adsorption	Chromium	[52]
36	MIL-53	Cr/Fe/Al	(O_2_C)-C_6_H_4_-(CO_2_)	Membrane Separation Adsorption	Dyes, Heavy metal	[53]
37	Ni-MOF-74	Ni	2,5-dioxide-1,4-benzenedicarboxylate	Adsorption, catalyst	CO_2_	[54]

ZIF is a subfamily of porous MOFs which uses imidazole-based ligand and transition series metal ion (Zn or Co). Their large surface area, adjustable surface characteristics, and chemical stability make them industrially most applicable. Mostly ZIF-based MOFs are water stable in nature. ZIF-8 retained its primary framework in boiling water for one week [18]. It is possible to explain ZIF-8’s exceptional stability via (a) Hydrophobic pores and surface topography which resist water molecules, and (b) Extremely stable covalent interaction between the metal ions (e.g., Zn^2+^ and Co^2+^) and imidazolate linker. ZIF-8 was prepared in the shortest time (approx. 5 min.) at room temperature with the highest product yield [58]. They also tuned ZIF-8 crystal size from micrometer (μm) to nanometer (nm), which also changed the morphology of crystals by using different capping agents like cetyltrimethylammonium bromide (CTAB). Some supplementary additives like sorbitan monooleate (Span 80), triethylamine (TEA), and poly (oxyethylene sorbitan monooleate), also known as Tween 80, are added to speed up the crystallization of ZIF in aqueous environments. For example, when TEA is used as a protonation agent, a group of researchers found the synthesis process of ZIF-67 and ZIF-8 in water within ten minutes at ambient temperature and atmospheric pressure [59]. Organic solvents are not environmentally friendly, expensive, and constitute a risk to the environment due to their toxic nature. Therefore, it is essential to create a novel and ecologically friendly synthesis technique that does not call for the use of potentially hazardous organic solvents [60]. Water has driven to be overdrawn this situation, as it is a most environmentally friendly solvent and readily available. As a result, it saves money and is more environmentally friendly than organic solvents. In comparison to organic solvents, water-based MOF synthesis improves material characteristics since organic solvents are locked in pores and difficult to remove compared to water removal. Presently, large numbers of MOFs are synthesized in water, such as ZIF, iso reticular MOFs (IRMOFs), MILs, UiOs, porous coordination network (PCN), and coordination pillared-layer (CPL) [61].

IRMOF series mostly uses dicarboxylic acid and tricarboxylic acid as organic linkers. The most widely studied IRMOF are MOF-5, MOF-177, MOF-74, and MOF-199 (HKUST-1 or Cu-BTC). HKUST-1 can also be synthesized in aqueous conditions. Huo et al. proposed a technique for synthesizing HKUST-1 at room temperature by combining metal salt powder of anhydrous copper (II) acetate (Cu(OAC)_2_) with excess ligand, i.e., H_3_BTC or 1,3,5-Benzenetricarboxylic acid only utilizing water as the reaction medium. [62].

MIL series are thermally highly stable classes of MOF. The MIL-53 series is commonly synthesized using a variety of metal ions (e.g., Sc, Fe, Ga, and Al) and linkers based on terephthalic acid, and their derivatives (e.g., fluorine, chlorine, amino, hydroxyl, nitro, and carbamate) as the organic linkers. Cheng and colleagues developed a simple solvothermal method for producing nanocrystals of NH_2_-MIL-53(Al) by modification of the water content in a DMF-water solvent mixture [63]. Other explored MOFs of the MILs family, including MIL-100, and MIL-53 may be synthesized in an aqueous solution.

UiO series have high thermally and chemically stable morphologies. The solvent DMF is used in the synthesis of the most widely studied UiO sequence, and Zr–MOF, which results in a significant amount of waste and by-products [61].

## 3. Biological Applications of MOF

MOFs could make a difference in biological applications for their suitable toxicology, acceptable stability, biocompatibility, biodegradability, and low cytotoxic effects.

### 3.1. MOFs for Biosensing

**Figure 4:**
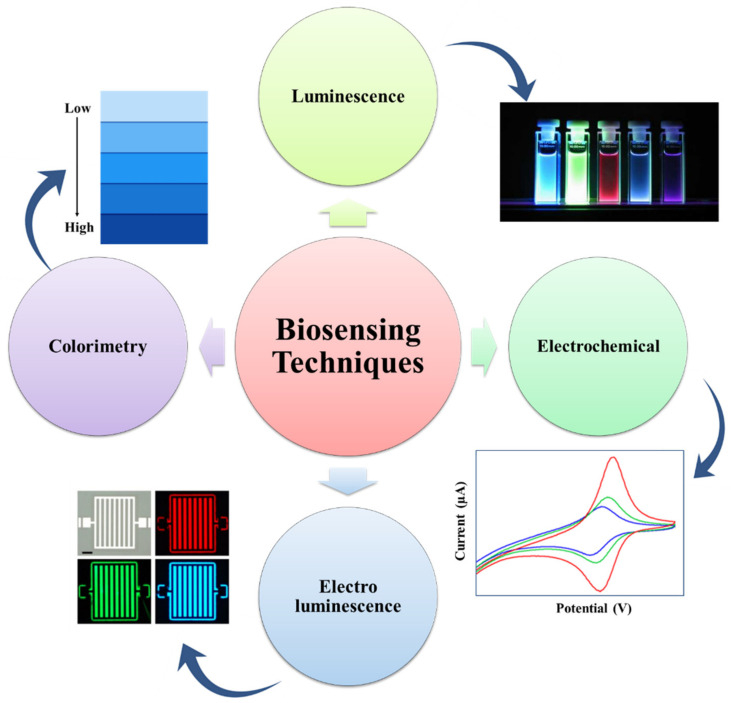
Representation of the many detection techniques utilized in the MOF-based biosensor for the sensing of biomolecules, bacteria, ions, and cells (adapted from [64]).

The biosensing characteristic can be achieved by a biosensor. A biosensor is a sensor that can determine the concentration of a biological analyte and quantify it. MOFs are intended to be a potential contender for electrochemical biosensors due to their high thermal, structural, and chemical stabilities. For biological sensing, biosensors should be extremely selective and sensitive. When compared to inorganic nanomaterials (e.g., gold nanoparticles, graphene oxide, and graphene), MOFs are biodegradable and have a low cytotoxicity, allowing for quicker degradation and the utilization of biocompatible building blocks. MOFs are utilized to detect RNA, DNA, enzyme activity, and small biomolecules. Magnetic resonance imaging (MRI) and computed tomography (CT) are critical clinical diagnostic procedures used to detect various diseases. Table 2 represents a list of MOFs with their biosensing applications. The biosensors detect the targeting agent based on the various techniques like electrochemical, colorimetric, luminescence, and electroluminescent methods, as represented in Figure 4 [64].

**Figure 5:**
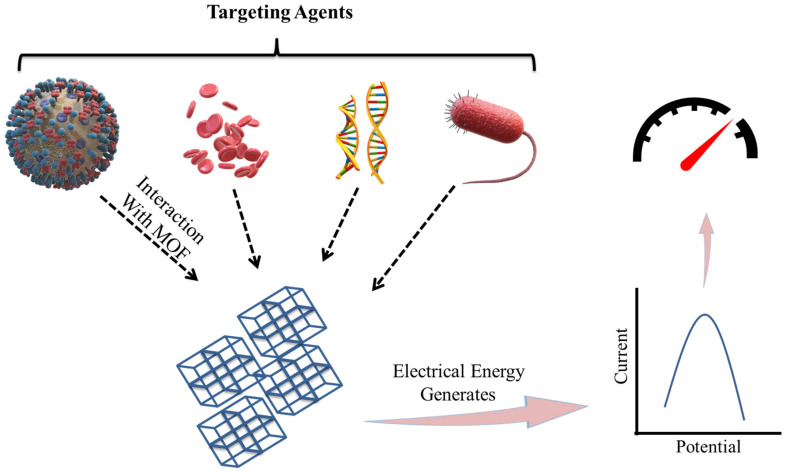
Diagrammatic representation of Electrochemical Sensing of targeting agents by MOF- based sensors (adapted from [74,75,76,77]).

Electrochemical method-based biosensors contains an electrode often known as a transducer. An electrochemical biosensors for pathogen detection is made up of conducting and semiconducting materials. An electrochemical process involving the electrode and an electrolyte solution of having pathogens converts the chemical energy involved in the binding process between the electrode-immobilized biorecognition components and target pathogens into electrical energy [74]. Energy will be detected by detectors, as shown in Figure 5 [74,75,76,77].

Colorimetric method-based biosensors are optical sensors, which change color when exposed to different stimuli. A stimulus can be defined as any physical or chemical change that generates optical signals [75].

Luminescence method-based biosensors have been reported for BL and CL detection using chemiluminescence, thermo-chemiluminescence (TCL), and electrogenerated chemiluminescence (ECL) reactions. They can be used for the measurement of images using chemical luminescence-based biosensors [76]. Basically, chemiluminescence is a luminescent signal process produced by an enzyme-labeled antibody, while electroluminescence is a luminescent signal produced by an electron transfer reaction between two luminescent compounds [77].

#### 3.1.1. Enzyme and Protein Biosensing

MOFs are biocompatible with proteins and enzymes and thereby achieve high enzyme/protein sensing. Zhang and their team developed a Zr-based PCN-222 photoelectrochemical (PEC) sensor for the detection of α-caesin. They estimated 0.13 µg/mL limit of detection (LOD) [78]. PCN-222 have a three-dimensional (3D) structure in which Zr metal is attached with tetrakis(4-carboxyphenyl)porphyrin (TTCP) ligand. Zhong Wei Jiang and coworkers used two-dimensional (2D) ytterbium-based MOF doped with gold nanoparticles (Au-NPs) as photoelectrochemical aptasensor for the detection of SARS-CoV 2 spike glycoprotein (S protein) with the detection limit of 72 ng/L [79]. Since 2D, Yb-TCPP shows good photoelectric performance and after the interaction with S protein, the photoelectric performance decreased [79]. Linjie Wang and coworkers synthesized a Zr-MOF for the colorimetric sensing of phosphorylated proteins as a mimic of peroxidase with the 0.16 µg/mL detection limit [80]. Synthesized MOF contains Zr as metal node, 2,2′-Bipyridyl-4,4′-dicarboxylic acid as a linker, and 3,3′,5,5′ -tetramethylbenzidine (TMB) as substrate. The presence of phosphorylated protein decreased the mimicking activity of Zr-MOF, and this suppressed activity is used in the quantitative determination of protein [80].

#### 3.1.2. DNA and RNA Sensing

Single-stranded DNA (ssDNA) quenched by virtue of fluorescent MOF (FMOF) utilizing fluorescence detection of nucleic acid (NA). A similar condition arises for HIV-1 DNA sequences and thrombin with Cu(H_2_DTOA) MOF. Virus detection relies on the identification of DNA and RNA; certain viruses have single-strain DNA (HIV ss-DNA, respiratory syncytial virus), double-strain DNA (HIV ds-DNA), and s-RNA (Ebola virus, zika virus), and they are also identified by MOFs [81]. Zhao et al., in 2016, reported Zn metal-based water- stable MOF for the sensing of HIV-1 ds DNA sequence [67]. They synthesized six water-stable MOF using N-(4-carboxybenzyl)-(3,5-dicarboxyl)pyridinium (Cbdcp) and zinc nitrate hexahydrate as basic constituents. Among them, {[Zn(Hcbdcp)_2_].H_2_O}_n_ binds with the P-DNA through hydrogen bonding or π-π stacking. This is an effective fluorescence probe for the detection of HIV-1 ds DNA, with detection limit of 10 pM [67]. In 2020, Lin and co-workers reported Cu-MOF for the sensing of Hepatitis B virus (HBV) with 5.2 fm limit of detection electrochemically [82]. For this sensing application, they used electroreduce graphene oxide (ErGO) as an amplifier which increased the electrochemical performance of Cu-MOF. The hybridization of probe ssDNA with ErGO-MOF decreased the current intensity by the increasing resistance in differential pulse voltammetry analysis [82]. Han et al., in 2022, reported a ratiometric DNA-functionalized MOF for the detection of cancer cells RNA, namely, miRNA-21 in living cells with the 57 pM detection limit [83]. Rahmati et al., in 2022, reported Ni_3_(BTC)_2_ MOF-based screen printed carbon electrode (SPCE) for the sensing of SARS-CoV-2, with 3.3 ± 0.04 PFU/mL and 20 min response time [84].

### 3.2. Biomedical Imaging of MOFs

**Figure 6:**
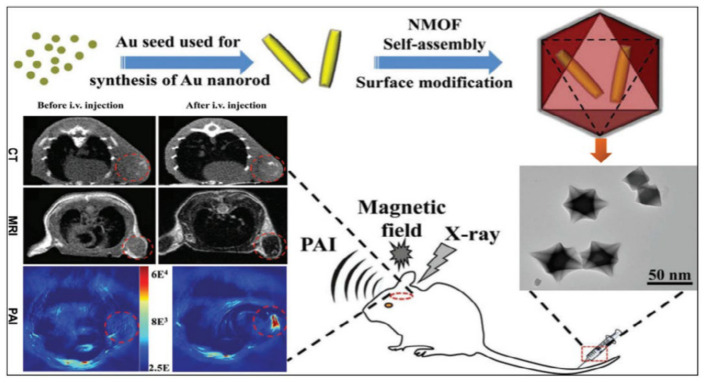
Schematic illustration of the synthesis of core-shell Au@MIL-88(Fe) nanostars and their in vivo triple-modal CT/MRI/PAI images of U87 MG-orthotopic tumor-bearing mice [87].

MOFs are used in biomedical imaging applications to make it easier to find and identify a number of disorders. MOFs are frequently applied to provide observable signals or to increase the contrast of particular tissues. This is usually achieved by changing the metal nodes of the MOFs. The most frequently used imaging techniques for MOFs are magnetic resonance imaging (MRI), positron emission tomography (PET), and computed tomography (CT). Fluorophores in MOFs have also made it possible for cells to use up conversion for optical imaging [85,86]. Glioma is one of the most common central nervous system tumors with high fatality rates. The spatial resolution, sensitivity, and penetration depth of glioma imaging is improved by integrating CT, MRI, and PAI with a nanocomposite made up of core-shell Au@MIL-88(Fe) [87], as shown in Figure 6.

#### 3.2.1. Intracellular RNA DNA Bioimaging

For intracellular DNA and RNA sensing, ultrathin MOF nanosheets associated with labeled probes give fluorescence quenching. For example, MicroRNA (miRNA) strategy consists of peptide nucleic acid (PNA) probes labeled with fluorophores and UiO-66 (nano MOF). This strategy has been investigated for multiplexed microRNA detection in live cancer cells. Here, nano MOF works as a fluorescence quencher and is labeled with different fluorescent peptide nucleic acid (PNA). After the modification, 10 pM LOD was reported for the (miR-21, miR-96, and miR-125b) detection. Importantly, the hybridization of PNA with miRNA causes the recovery of fluorescence. [88]. More recently, 2-D Ultrathin MOF-La nanosheet, i.e., {[La_2_(TDA)_3_]_2_H_2_O}n has been reported as a sensing platform for DNA sensing and ratiometric monitoring of adenosine in single cells [85]. The potentially ratiometric biosensing is possible due to fluorescence quenching, where quenching occurred on La-MOF through charge transfer from dye molecules to the La^3+^. Interestingly, charge properties in view of positive or negative effects of the labeled fluorophores have been utilized for ‘‘turn-down followed by turn-down” and ‘‘turn-down followed by turn-up” process for DNA sensing [85].

#### 3.2.2. MR Imaging (MRI)

The goal of this method is to find nuclear spins that have changed orientation in a magnetic field. It easily picks up signals from the many hydrogen atoms in biological systems by using radio waves and an external variable magnetic field. MRI does not require ionizing radiation, thus preferred over CT. These signals provide detailed anatomical maps that aid in the diagnosis of diseases and other anomalies.

MRI is superior to any optical imaging technique in terms of penetration depth confront [89]. The magnetic relaxivity enhances the MRI image contrast. It is given as a concentration-normalized change in the transverse or longitudinal relaxation rate (1/T1 or 1/T2) per millimole of contrast agent (mM-1s-1).34 Gd-based MOFs have developed into a promising option for MRI contrast agents as gadolinium-based small molecules are most extensively utilized as contrast agents [86]. There are several experiments reported related to the Gd-containing nano MOFs [90]. The relaxation values of these Gd-containing nanoscale MOFs were a considerable level greater than the extensively utilized clinical contrast agent such as Omni scan [91]. Encapsulation of superparamagnetic nanoparticles in MOFs may provide effective contrast agents as an alternative method [92]. As a platform for MRI-guided photothermal treatment, Gd-DTPA-grafted MOF-808 nanoparticles with a polyaniline (PANI) surface modification are utilized [93]. The resultant MOF has a high longitudinal relaxivity (30.1 mM^−1^s^−1^) that is 5.4 times higher than other contrast agents [93]. To overcome Gd’s toxicity, several researchers are now concentrating on alternate metal-based MOFs. Yang and coworkers created Mn^+2^- based MOFs from dyes of organic ligands which can adsorb near-IR that served as MRI contrast agents [94]. In another study, Chowdhuri and co-workers developed MOFs with internalized Fe_3_O_4_ nanoparticles as MRI contrast agents [95]. Additionally, MRI contrast agents with Fe-based MOFs (MIL-53-Fe) containing anticancer drugs or oligonucleotides, such as Fe-MIL-53, have been investigated [96]. Mn(II)- and Gd(III)-based MOFs, namely {[Mn_2_(Cmdcp)_2_(H_2_O)_2_]·H_2_O}_n_ and {[Gd(Cmdcp)(H_2_O)_3_](NO_3_)·3H_2_O}_n_ from a zwitterionic carboxylate ligand were synthesized by Quin et al. in 2017 [97]. Good relaxivities, T_1_-weighted pictures, and low impact on a kidney cell line (human embryonic) are all characteristics of both MOFs. For a prolonged period of time, these MOFs captured high-resolution MRI images with high contrast efficiency [97].

#### 3.2.3. X-ray Computed Tomography Imaging

Computed tomography (CT) imaging, or CT scanning is a method that generates images of a subject’s internal anatomy using the attenuation of X-rays. An item is exposed to X-rays from a variety of angles, and a collection of cross-sectional images is merged to form a 3-D image. Usually, high atomic number elements such as I, Ba, and Bi are utilized. The usage of MOFs is favorable due to the presence of element with higher atomic number as metal nodes. For example, Zhang and co-workers developed a UiO-PDT framework which contains BODIPY. In vivo CT imaging reveals that MOF nanoparticles preferentially accumulate at tumor locations, hence contrast increased [98,99] over conventional contrasting agents. Sheng and co-workers reported a MIL-88 MOF connected with gold-nanoparticles utilized as a multipurpose diagnostic tool for excellent CT scans [100].

#### 3.2.4. Positron Emission Tomography (PET) Imaging

At the organs of interest, Positron Emission Tomography (PET) uses radionucleotides that release positrons and break down into gamma ray photons that can be detected. The detector aggregates data to create a three-dimensional picture. Quick imaging speed, great sensitivity, deep penetration, and excellent quantitative capabilities make PET imaging the best of all imaging techniques [101]. MOFs containing radioisotopes are suitable for positron imaging technique. A MOF-based material may have been used to provide safe and stable nanoplatforms for PET imaging [101]. A radioactive UiO-66 was synthesized which contains ^89^Zr metal nodes in their structure. It was activated by using pyrene-derived poly(ethylene glycol) (Py-PGA-PEG) and long peptide ligands [98,102]. They functioned as tumor-selective PET imaging agents (in vivo) and showed strong material and radiochemical reliabilities in numerous biological situations. The literature states that ^89^Zr has a half-life that is substantially longer (78 h) than the conventionally used ^19^F (2 h) [98]. ^89^Zr with high half-life usage was used to monitor the clearance and distribution processes of ^89^Zr-UiO-66/Py-PGA-PEG-F_3_ in vivo for up to 120 h after intraperitoneal administration [101]. Lu et al. studied the toxicity estimation for ^89^Zr-UiO-66, which was not toxic, even when it was applied at a dose of 50 mg/kg for 1 month, which was caused in the experimental groups [103].

The half-life of the positron emitter ^64^Cu is 12.7 h, and it is a radionuclei with ideal decay characteristics for imaging nanomedicine in vivo using PET. The ^64^Cu radiolabeling technique without chelators was created by the researcher for tracking MOFs in vivo condition. The reason for tracing MOFs is to study the drug delivery distribution and excretion pattern in subject models [104]. Cu-labeled MOF-Au-PEG was used in the investigation of nanomedicine biodistribution in tumor-bearing female mice PET imaging [105]. Table 3 shows the names of different metal organic frameworks used in the bioimaging application.

### 3.3. MOF as a Drug Delivery System (DDS)

**Figure 7:**
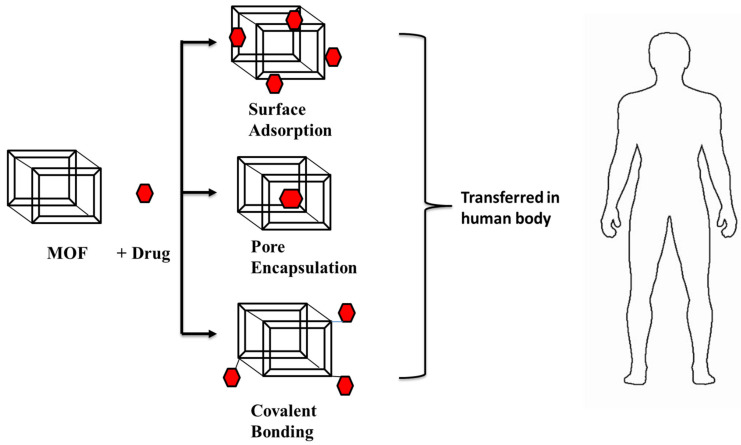
A schematic representation of the Drug delivery system in the human body (adapted from [113,114,115]).

DDS is a dynamic biological subject within material science that has a widespread application in human health. When compared to other porous materials, MOFs are a good candidate for drug delivery because of their highly tunable nature (pore size as well as tuning of the metal ion or organic linker), large surface area, and pore size [112]. Nano-MOFs, which were created by scaling down MOF particle size, are effective for drug delivery vectors. In the last decade, they have been a focal point in the area of drug delivery devices for distributing the loaded drug to specified places. Among the reported porous carriers, MOFs gained attention as they had desired characteristics such as having a large cavity size for drug encapsulation, exceptionally high surface area, and a controlled drug-release profile. They exhibited inherent biodegradability and varied functionality for post-synthetic grafting of medicinal molecules because of their metal-ligand interactions, which are relatively labile in nature [113]. A variety of hydrophobic, hydrophilic, and amphiphilic medicinal molecules could be encapsulated in the cavity of MOF and/or attached to the framework structures [114]. Drug loading in MOFs is achieved through covalent interactions or non-covalent interactions, as shown in Figure 7 [113,114,115]. Drug molecules that are covalently attached to MOFs can release drugs more slowly than drug molecules that are just stuck to their surfaces [114]. Physicochemical properties of MOF materials and the drug molecule (3D arrangement, pore size) are two things that affect how MOFs are used to deliver drugs. It allows the drug molecules to fit within the carrier molecules so that they can easily reach their target. In the case of nanocarriers, burst release of drug molecules were observed. However, release of drug molecules from MOFs is delayed and regulated by matrix breakdown [115]. For example, iron-containing BioMIL-1 MOFs displayed greater nicotinic acid loading (up to 75%) than the native MOF structures and regulated drug delivery [116].

To make its toxicology feasible for MOFs in their biological applications, suitable metal ion and organic linkers with the lowest cytotoxicity level are desired. The best suitable metals are Cu, Mg, Ca, Mn, Fe, Zn, Ti, and Zr (a non-toxic carrier is a prerequisite to every drug). The biodegradability and stability of MOFs is another contentious issue pertaining to their application in drug delivery systems since it facilitates drug diffusion from matrix materials, hence enhancing their drug release efficiency.

MOFs adsorb relevant substances on their exterior surface, channels open, or capture molecules within the frameworks. Additionally, active molecules may possibly be introduced into MOFs via covalent bonding by either a post-synthetic modification or one-pot synthesis [112]. Differences from functionalizing MOFs with therapeutic agents for biological applications are as follows:

- Surface Adsorption

MOFs are capable of adsorbing functional molecules due to their large surface area and porosity. Most of the time, surface adsorption is conducted by stirring MOFs that have already been made in a solution with functional molecules. Hydrogen bonding, Van der Waals interaction, and π–π* interaction, are the key forces involved in this method. Surface adsorption has been extensively used to immobilize enzymes [117]. In 2006, the Balkus group showed that a microperoxidase-11 (MP-11) catalyst could be physically attached to a MOF during keeping the catalytic performance of the MP-11 catalyst, which contains Cu as metal and nano-crystalline in nature [118].

- Pore Encapsulation

The pores of MOFs may accommodate a wide variety of functional molecules because of their high porosity and pore adjustable properties, which range from microporous to mesoporous. Anticancer drugs are encapsulated within the host of the MOF for later intracellular uptake and release [112]. For instance, encapsulation of camptothecin was performed using ZIF-8 nanospheres with 70 nm particle size [119].

- Covalent Binding

The methods described above rely on very weak interactions between molecules and MOFs, which frequently results in delayed leaching difficulties. Functional groups present on the MOF surface, such as carboxyl, amino, and hydroxyl groups, establish covalent interactions with active groups onto the target [112]. Jung et al. revealed the post-synthetic conjugation of Candida Antarctica lipase B (CAL-B) and increased green fluorescent protein (eGFP) on the MOF surface [120].

- Functional Molecules as the Building Block

Using functional molecules as building blocks is another option. Generally, biomolecules include a number of types of reactive chemicals that are compatible with inorganic metals. Until now, amino acids [121], peptides [122], nucleobases [123], and saccharides [124] might function as organic ligands. These biomolecules are used in the synthesis of bioMOFs. BioMOFs often exhibit superior biocompatibility and unique biological functioning. By mixing zinc acetate dihydrate, adenine, and biphenyl dicarboxylic acid (BPDC), the research team made bioMOF-1, which is crystalline and porous in nature. One of the commonly used chemotherapy agents for ovarian cancer, breast cancer, and lymphoblastic leukemia is doxorubicin hydrochloride (DOX) [125]. As an alternative to whole-body radiation therapy for leukemia, the amphiphilic anticancer agent busulfan (Bu) is frequently utilized in chemotherapy [126]. Topotecan (TPT), which is made up of a camptothecin (CPT), is therapeutically used to treat small cell lung cancer and refractory ovarian cancer [127,128]. ZIF-8 is a MOF which contains zinc as metal node and 2-methylimidazolate as linker. Due to its superior hydrothermal stability, thermal stability, biocompatible qualities and non-toxicity, ZIF-8 is being identified as a possible nanocarrier to use in drug delivery [129]. Notably, in physiological conditions, ZIF-8 is stable, but in acidic environments, it is unstable. Therefore, ZIF-8 is used in pH-sensitive methods related to drug delivery. After its synthesis in 2012, a group of researchers effectively loaded DOX (4.9 wt%) by mixing of ZIF-8, followed by the addition of dry ZIF-8 powder to aqueous medium [130]. This results in being extremely regulated, and after 30 days, 66% drug release were observed. Similarly, ZIF-8 was employed as a pH-responsive drug delivery channel in the case of 5-fluorouracil (5-FU) delivery [131].

**Figure 8:**
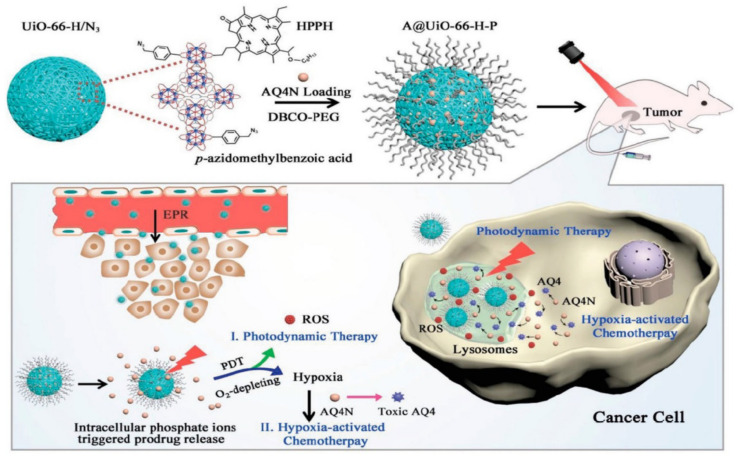
Synthetic procedure of A/UiO-66-HP nanoparticles and mechanism of photodynamic therapy and hypoxia-activated cascade chemotherapy [132].

The hypoxia-activated prodrug banoxantrone (AQ4N) was loaded onto UiO-66 MOF, and during the manufacture of UiO-66 nanoparticles, p-azido-methyl benzoic acid and monocarboxylate photo color were used as modulators to modify MOF with the photosensitizer, photo color (HPPH), and azide groups (N_3_). This is used for hypoxia-activated cascade chemotherapy [132]. Figure 8 shows the procedure used for the synthesis of A/UiO-66-HP nanoparticles, photodynamic therapy mechanism involved, and hypoxia-activated cascade chemotherapy.

In addition to the above examples, Table 4 listed some other MOFs used in the drug delivery system. Additionally, MOFs are capable of molecular recognition, which is useful for sensing applications. The literature demonstrates that using biomolecules as an organic linker in MOFs increases their potential for application in biosensing, biocatalysis, imaging, and other applications.

### 3.4. Miscellaneous Biomedical Applications of MOFs

Despite the biosensing, bioimaging, and drug delivery, MOFs are also used as the antitumor agent. MOFs are also used in radiotherapy as well as in chemodynamic therapy [148]. In 2018, Kaiyuan Ni and their team reported two hafnium-based MOFs, i.e., Hf_6_-DBA and Hf_12_-DBA, for the radiotherapy [149], where DBA stands for 2,5-di(p-ben-zoato)aniline). The SBU unite of these MOFs generates reactive oxygen species (ROS) after the absorbance of X-rays, which results in the higher radioenhancing efficiency. To find out the efficiency of these MOFs, they examined apoptosis and DNA double-strand break (DSB) pathways. After their investigation, they found Hf_12_-DBA was found more superior than Hf_6_-DBA for radiotherapy [149]. In 2021, Prajapati and coworkers used CuSO_4_ as metal and L-cysteine as a linker for the synthesis of a metal organic hybrid CuHARS for the treatment of glioma cells [150]. In an another study, polyallylamine hydrochloride (PAH)-coated CuHARS and cellulose fiber are used for the degradation of S-nitrosothiol for the antimicrobial activity [151]. Healing of wounds is also a major concern for diabetic patients. For this, in 2017, Jisheng Xiao and coworkers reported the wound-healing property of a HKUST-1 after the integration with citrate-based hydrogel. The integration of hydrogel accelerated the wound-healing capacity as well as decreased the toxicity generated by copper metals [152]. Yao et al., in 2020, used ZIF-8 MOF for the wound healing. They loaded omniphobic porous gel with ZIF-8 and used it as a wound healing material. Omniphobic ZIF-8@hydrogel porous wound dressing can prevent bacterial growth and enable the regulated release of the bactericidal, anti-inflammatory, and non-toxic zinc ions for wound healing [153].

## 4. Critical Issues and Role of MOFs in Biological Applications

### 4.1. Role of Synthesis Techniques

MOFs may serve as precursors for the synthesis of metal oxide-embedded carbon. Versatile nitrogen-doped carbon hetero structures were produced through hybrid coating of ZIF-8/ZIF-67 on cotton [154]. The materials can be utilized for electrocatalytic oxygen reduction, supercapacitors, and electromagnetic interference shielding, among many other uses. When the Zn/Co bimetallic ZIF-8/67 was heated to 900 °C, the presence of cellulose stopped catalytically active Co nanoparticles from sticking together [154].

Many synthesis techniques for MOFs have been developed by a keen choice of metal ions/organic linkers and aqueous solvent systems. Basically, MOFs have been synthesized using various combinations of metal ions (Cu^2+^, Zn^2+^, Fe^3+^, Mn^2+^, Co^2+^ Ni^2+^ Cr^2+^, and Ag^+3^) and a family of biological linkers (amino acids, peptides, proteins, nucleobases, and saccharides) or any suitable organic linker for biological applications. MOFs are synthesized using various methods such as one-pot self-assembly, hydrothermal/solvothermal, microwave, electrochemical, mechanochemical, sonochemical, slow diffusion of reagents, precipitation, etc. [155]. The synthesis method/approach for MOFs is chosen based on major facts, such as the solvent system, the type of organic linkers selected, the type of biomolecules selected, and the targeted applications. Hydrothermal/solvothermal, micro precipitation, and slow diffusion are common synthesis methods/approaches that are widely used for MOF synthesis. In the following section, we will explore these minor points in light of the latest report and attempt to ascertain the novel possibilities and obstacles associated with commercial biological uses of MOFs.

### 4.2. Role of Organic Linkers

MOFs are constructed using biomolecules or bioligands or natural/bioinspired organic ligands having good coordination capabilities with selected or specified metal ions. A variety of biomolecules, such as amino acids, proteins, peptides, nucleobases, carbohydrates, porphyrins, and polyhydrins are being used as ligands for synthesis of MOFs/BioMOFs. These bioligands influence a strong effect on flexibility, structural robustness, and physical properties. Advantages of these ligands include their commercial and natural availability, straightforward synthetic methods for the synthesis of novel ligands, structural diversity, various metal-binding sites, and inherent chirality.

#### 4.2.1. Amino Acids, Peptides, and Protein

Amino acids are biomolecules containing both amine (-NH_2_) and carboxyl functional groups, which determine their chemical/physical properties. The amino acid possesses a polar group as well as non-polar side chains. Consequently, coordination with the amino acid-derived MOFs possesses chirality and is utilized for specific and selective sensing and separation. Various BioMOFs (like MOF-11, NH_2_-MIL-101(Al), NH_2_-MIL-53(Al), and NH_2_-MIL-101(Cr) have been synthesized and reported for biological applications [156,157,158,159].

So far, we have observed that the synthesis of BioMOFs is preferably achieved by post-synthetic treatments. As MOFs possess a large surface area and high functionality, several types of bioconjugation techniques can be applied for the development of different sensing applications. The biomolecules can be impregnated into the MOFs via diffusion, surface immobilization, encapsulation, biofunctionalization, and various other methods. For example, Zn (Gly-X)_2_; frameworks, ZnGGH-1.(DMF-H_2_O) and the DOX- encapsulated MOF paired with a targeting peptide (RGD and AP2H) can be promising as a targetable delivery system for cancer treatment [160].

Similarly, peptides are biomolecules composed of chains of amino acids that are linked by amide bonds. These represent a family of peptide species that can be created by altering the amino acid sequence and type. Peptides are critical structural components of living beings and are regarded as the primary antecedents to life. Dipeptides are the shortest polypeptides and are utilized extensively in the manufacture of MOFs/BioMOFs. For example, β-alanyl-l-histidine (carnosine dipeptide) was used as a bioligand in conjunction with Zn(ll) metal ions to create a water-stable ZIF-type MOF [122]. Tripeptides such as Gly-l-His-l-Lys (GHK) and Gly-l-His-Gly (GHG) were coordinated with Cu(ll) metal ions to generate two iso reticular 3D peptide-based porous BioMOFs with larger pores, e.g., CullGHG and CullGHK. These BioMOFs possess sponge-like behavior, thus show reversible behavior and collapse upon evacuation to come back to the original structure by exposing the MOF [161]. There are very few polypeptides MOF/BioMOFs than dipeptide BioMOFs to the best of our knowledge. The reason behind this is the increase in flexibility of long-chain polypeptides and difficulty in forming 3D frameworks. Apart from this, the instability of these sorts of MOFs caused by the presence of considerably larger groups of amino acids may result in interpenetration and disruption of the MOF’s pore size. However, the adaptability and dynamic responsiveness of peptides to a guest molecule are ensured by their flexibility.

There are many advantages while using proteins as ligands in the synthesis of MOFs such as more coordination sites leading to structural diversity and various physical and chemical properties [162]. Protein-derived MOFs can be used as a biocatalyst in important biological processes in the body [9]. Several natural proteins known to date require the binding of particular metal ions at a specific position. Metals are required for protein function, but proteins have a complex and flexible structure, and it is very hard to control how metal ions are arranged on their surfaces. Hence, it is difficult to successfully design and produce protein MOFs/BioMOFs. Protein crystalline frameworks (PCFs) were synthesized using histidine like His59, His63, His73, and His77 with metal ions such as Zn, Cu, or Ni [162,163]. Bailey et al. (2017) constructed 15 ferritin protein MOFs (PMOFs) using ferritin nodes (Ni (II), Zn(II), and Co(II)) and synthetic di hydroxamate linkers as metal ions and organic linkers, respectively [164]. The expected lattice arrangement was body-centered (cubic and tetragonal). According to the small-angle X-ray scattering (SAXS) analysis, these PMOFs were suggested to adopt multiple lattice confirmation to support dynamic behavior [164]. Recently, Protein@ZIF-8 biocomposites were also prepared in aqueous condition at room temperature using horseradish peroxidase (HRP), bovine serum albumin (BSA), trypsin (TR), alcohol oxidase (AOX), hemoglobin (HGP), and myoglobin (MB) [165]. The MOF-based biocomposite was created within a few minutes after Zn^2+^ and 2-methylimidazole (mIM) were added to the mixture of biomacromolecules at room temperature in an aqueous medium [166].

#### 4.2.2. Miscellaneous

The integration of functional groups on MOFs should exert dominant controls on the creation of new multifunctional composite and hybrid materials. These modified materials are likely to exhibit unique properties, which will make these materials superior to their pristine forms with enhanced and improved synergizing effects. For example, Rh@BioMOF-l composites were synthesized via post-synthetic modification (PSM) of BioMOFs. The method for PSM involved the soaking of BioMOF-1 in Rh (rhodamine) dye solution, which led to the coloring of MOF particles to confirm that dye molecules were hosted by the BioMOF-1 [167]. In another report photo, functional hybrid Tb^3+^@BioMOF-1 was synthesized through the post-synthetic method via cation exchange [168]. Luminescent hybrid MOF has quenched the fluorescence response while interacting with oxygen.

MOFs, as opposed to classical coordination chemistry, are created from multifunctional ligands that allow complex formation in a repeated way, a process known as supramolecular polymerization (polymerization templating) in three dimensions [169]. Considering that the advantageous characteristics of both material classes may be integrated in this fashion, combining MOFs with polymers appears to be a potential option for a composite or hybrid material. Polymers bring processability, mechanical/chemical durability, and applicability in the biomedical field, whereas MOFs have well-defined porosity, metal content, and specified crystal structures. These characteristics make MOFs ideal candidates for biomedical applications like drug delivery and magnetic resonance imaging (MRI) [92,170,171].

Through the coordination of amine-bearing polymers to the Iron(III) ions of the MOF, Horcajada et al. engineered the surfaces of iron(III) carboxylate MOF nanoparticles. In particular, alpha monomethoxy-omega-amino poly(ethylene glycol) (CH3-O-PEG-NH2) was added during the synthesis of MIL-88A and MIL-89 to provide the matching PEGylated nanoparticles. In a similar manner, chitosan-modified MIL-88A particles were synthesized utilizing chitosan grafted with lauryl side chains. PEG chains were also used to post-synthesize MIL-88A and MIL-100, as well as dextran-fluorescein-biotin. Azidothymidine triphosphate was impregnated into MIL-88 and MIL-100 PEGylated nanoparticles before being tested for HIV activity. In aqueous conditions, it was found that the polymer coating inhibited the aggregation of pure MOF nanoparticles without altering the therapeutic outcomes. PEGylated nanoparticles had marginally higher transverse relaxivities than the non-PEGylated ones when tested as MRI contrast agents [7].

## 5. Opportunities and Challenges

Metal-organic frameworks (MOFs) are a good choice in the area of biosensors, bioimaging, and drug delivery systems because of their high thermal, structural, and chemical stabilities, biodegradable nature, minimal cytotoxicity, and ability to use biocompatible building components. Due to the variable pore size and high porosity of MOF, encapsulation, covalent binding, and surface adsorption of different drugs, proteins, and other functional molecules have been successfully realized with great efficiency. Fluorophores are used, and the metal nodes of MOFs are modified. Some of the most often utilized MOF-based imaging modalities include MRI, CT, and PET.

To avoid adverse reaction risk, more clinical studies need to be done for toxicology studies and biocompatibility of MOFs with a therapeutic drug. The therapeutic drug delivery system should consider metal ion/organic linkers with minimum toxicity. Moreover, biomedical applications can be enhanced by tuning the properties like pore size, and structural and functional changes in MOFs.

The low stability of Zn-carboxylate MOFs in aqueous solutions (due to low coordinative affinity) has restricted its biomedical applications. Hence, biostability of MOFs is very necessary under any physiological conditions. Drug delivery of therapeutic proteins with a low molecular weight (<7 kD) are easily filling up within the MOF molecules [172]. However, proteins with a greater than 10 kD molecular weight often require either large pores or channels to be loaded into the MOF. Hence, it is required to develop MOFs with large pore sizes for therapeutic protein delivery systems.

The main aim of this review paper is to present a comprehensive and critical overview of MOFs concerning their biological applications. The first section of the review gives a brief overview of water-stable and water-soluble MOFs, as well as a concise overview of their morphology, design, and synthesis. Following that, contemporary examples of MOFs, composites, and hybrids were addressed in order to give readers clarity regarding biomedical or biological applications. The foremost focus of this paper is on the future of biological-sensing applications using MOFs, as well as the opportunities and obstacles in this research field for real-world applications. This review, to the best of our knowledge, focuses on the analytical evaluation of MOFs in terms of biological use. Furthermore, this review article may serve as a catalyst for a number of scholars to pursue careers in this new field of research, with the interpretation, as well as the experimental conclusions that can be drawn.

## Figures and Tables

**Table 2:** List on various biosensing applications with different techniques by MOFs.

S. No.	MOF Formula	Quality of MOF	Metal Ion	Ligand	Targeting Agent/Detection	Source of Targeting Agent	Work Mechanism	Method	Detection Limit	Reference
1.	[Cu(dcbb)_2_ (H_2_O)_2_]∙ 10H_2_O}n	Water stable	Cu	1-(3,5-dicarboxybenzyl)-4,4′-bipyridinium bromide)	miR-185, miR-20a, miR-92b, miR-25 miR-210	Plasma of gastric carcinoma patients	Electrostatic/π-stack- ing interactions	Fluorescence quenching	172 ± 5 pM 321 ± 8 pM 91 ± 7 pM 91 ± 7 pM 132 ± 12 pM	[65]
2.	{[Al_4_(OH)_4_ (H_2_O)(NTB)_2_(HCOO)_3_] (HCOO) (NMF)14.5 (H_2_O)_4_}n	Water stable	Al	4,4′,4″-nitrilotribenzoic acid	Vomitoxin	Wine	NA	Electrochemical techniques	0.70 pg mL^−1^	[66]
3.	{[Al_4_(OH)_4_ (H_2_O)(NTB)_2_(HCOO)_3_] (HCOO) (NMF)14.5 (H_2_O)_4_}n	Water stable	Al	4,4′,4″-nitrilotribenzoic acid	Salbutamol	Pork	NA	Electrochemical techniques	0.40 pg mL^−1^	[66]
4.	{[Zn(Cbdcp)(H_2_O)_3_]·H_2_O}n	Water stable	Zn	{Na_3_[Na_9_(Cbdcp)_6_(H_2_O)_18_]}	HIV ds-DNA	NA	Electrostatic, π-stacking and/or hydrogen bonding interactions	NA	10 pM	[67]
5.	{[Dy(Cmdcp)(H_2_O)_3_](NO_3_)·2H_2_O}n	Water stable	Dy	Zwitterionic carboxylate ligand	Ebola virus RNA sequences	NA	Electrostatic, π -stacking and/or hydrogen bonding interactions	Fluorescent detection	160 pM	[68]
6.	Zn-BDC-TED	NA	Zn	Terephthalic acid and triethylenediamine	C-reactive protein (CRP)	Human serum	conductivity	Electrochemical immune sensor	5.0 pg/mL	[69]
7.	Zr-MOF:Eu^3+^	NA	Zr and Eu	Benzene tetracarboxylic acid	Bilirubin	Human serum	Fluorescent resonant energy transfer	Fluorescent probe	1.5 μM	[70]
8.	NH_2_-MIL-53(Fe)	Water dispersible	Fe	2-aminobenzene-1,4-dicarboxylic acid	Staphylococcus aureus	Pastry cream	NA	Photoluminescence	31 CFU/mL	[71]
9.	zirconium-porphyrin MOF (PCN-222)	Highly stable	Zr	meso-tetra(4-carboxyphenyl)porphyrin	chloramphenicol	Milk, shrimp	π-π stacking interactions	Ratiometric fluorescent sensing	0.08 pg/mL	[72]
10.	Cu-hemin MOFs	NA	Cu	Hemin	Glucose	Human serum	NA	Electrochemical sensor	2.73 μM	[73]

**Table 3:** List of metal organic frameworks used in the bioimaging applications.

S. No.	MOFs	Metal	Ligand	Integration Method	Function	References
1	NH_2_-MIL-53	Fe^3+^	2-Amino-terephthalic acid [H_2_NC_6_H_3_-1,4-(CO_2_H)_2_]	Surface amendment and encapsulation	Magnetic resonance imaging (MRI) of target cell	[96]
2	PCN-TTA-UC	Al^3+^	4,4′-(9,10-Anthracenediyl) dipyridine [C_24_H_16_N_2_]	Triplet-triplet annihilation alteration	Bio-imaging (In vivo)	[106]
3	UiO-66	Zr^4+^	Terephthalic acid [C_6_H_4_(CO_2_H)_2_]	BODIPY attachment	Computed tomography (CT) scan	[99]
4	UiO-66	^89^Zr	Terephthalic acid [C_6_H_4_(CO_2_H)_2_]	Radioactivity as secondary binding unit	Photodynamic therapy (PET) scan (In vivo)	[102]
5	Gd-MOFs	Gd^3+^	5-bromobenzene 1,3-dicarboxylic acid [C_16_H_10_Br_2_O_8_]	Encapsulation	Computed tomography (Chemotherapy)	[107]
6	TTA-UC MOF	Zr^4+^	4, 4′-Bis (alpha, alpha′-dimethylbenzyl) diphenylamine [C_30_H_31_N]		Optical bio-imaging	[106]
7	ZIF-8	Zn^2+^	Imidazole [C_3_H_4_N_2_]	Encapsulation	NIR response to cell	[108]
8	PPy@MIL-100(Fe)	Fe^3+^	Trimesic acid [C_6_H_3_(CO_2_H]	Micro emulsion encapsulation	Near infrared imaging (NIR) (T_2_ cancer cell)	[96]
9	C-dot@ ZIF-8 (nUiO-67)-[Ru(bpy)_3_]^2+^	Zn^2+^	2,2′-Bipyridine [C_10_H_8_N_2_]		Optical imaging	[109]
10	NMOF-1	Tb^3+^	1,3,5-Benzene tricarboxylate (BTC) [C_9_H_6_O_6_]	Luminescent surface modification	Magnetic resonance imaging	[7]
11	Gd-NMOF	Gd^3+^	1,2,3,4,5,6-Cyclohexanehexacarboxylic acid [C_12_H_12_O_12_]		Magnetic resonance imaging	[90]
12	Cu-NMOF	Cu^2+^	2,3,5,6 tetra-iodo 1,4-benzenedicarboxylic acid[C_8_H_2_I_4_O_4_]	Encapsulation	Computed tomography	[110]
13	Fe-MOF	Fe^3+^	1,3,5-Benzene tricarboxylate (BTC) [C_9_H_6_O_6_]		Magnetic resonance imaging	[7]
14	Fe-NMOF	Fe^3+^	2-Amino-terephthalic acid [H_2_NC_6_H_3_-1,4-(CO_2_H)_2_]	Surface modification	Optical imaging	[7]
15	Mn-MOF	Mn^2+^	1,3,5-Benzene tricarboxylate (BTC) [C_9_H_6_O_6_]		Magnetic resonance imaging	[111]

**Table 4:** List of MOFs used in drug delivery system with their targeting agent and drug loading percentage.

S. No.	Name of MOFs	Name of Drug	Biological Test System	Mechanism	Drug Loading %	References
1	ZIF-8	Doxorubicin	Breast cancer cell lines	Encapsulation	20% wt	[133]
2	ZIF-8	Ceftazidime	*Escherichia coli*	NA	~10.8% wt	[134]
3	MIL-100(Fe)	Indocyanine green	MCF-7 cells/xenograft tumors	π–π interaction	40% wt	[135]
4	MIL-100 (Fe)	Doxorubicin	HepG-2 cells	NA	29% wt	[136]
5	MIL-100 (Fe)	Metformin hydrochloride	PBS Buffer	pH-cleavable bonds	35% wt	[128]
6	MIL-101 (Fe)	BODIPY	HT-29 human colon adenocarcinoma cells	NA	11.6 wt %	[137]
7	MIL-101 (Fe)	Doxorubicin	H-22 tumor-bearing mice	NA	82.2% wt	[138]
8	MOF-74 (Fe)	Ibuprofen	PC12 cells	Ion exchange	15.9% wt	[139]
9	HKUST-1	Ibuprofen, anethole and guaiacol	PBS buffer	NA	0.34 g/g, 0.38 g/g and 0.40 g/g	[140]
10	MIL-100(Fe)	Doxorubicin	Tris Buffer	Host–Guest Interactions	9% wt	[141]
11	NU-1000	Insulin	Nucleic acids	NA	34% wt	[142]
12	NU-1000	Insulin	PBS Buffer	NA	40% wt	[143]
13	Zn-MOF	5-Fluorouracil	PBS Buffer	pH-controlled	44.6% wt	[144]
14	UiO-66@ Fe_3_O_4_	Doxorubicin	3T3, HeLa	π–π interaction	66.3% wt	[145]
15	UiO-66	Cisplatin	HSC-3 and U-87 MG cancer cell	Encapsulation	48 mg/g	[146]
16	UiO-68	Cisplatin	SKOV-3 cells	Encapsulation	2.3 ± 1.2 wt%	[147]
